# Supplementary figures and images for: Loss of Cdc13 causes genome instability by a deficiency in replication-dependent telomere capping
Source: PLoS Genet. 2020 Apr 14;16(4):e1008733. doi: 10.1371/journal.pgen.1008733 (PMC7205313; doi:10.1371/journal.pgen.1008733)

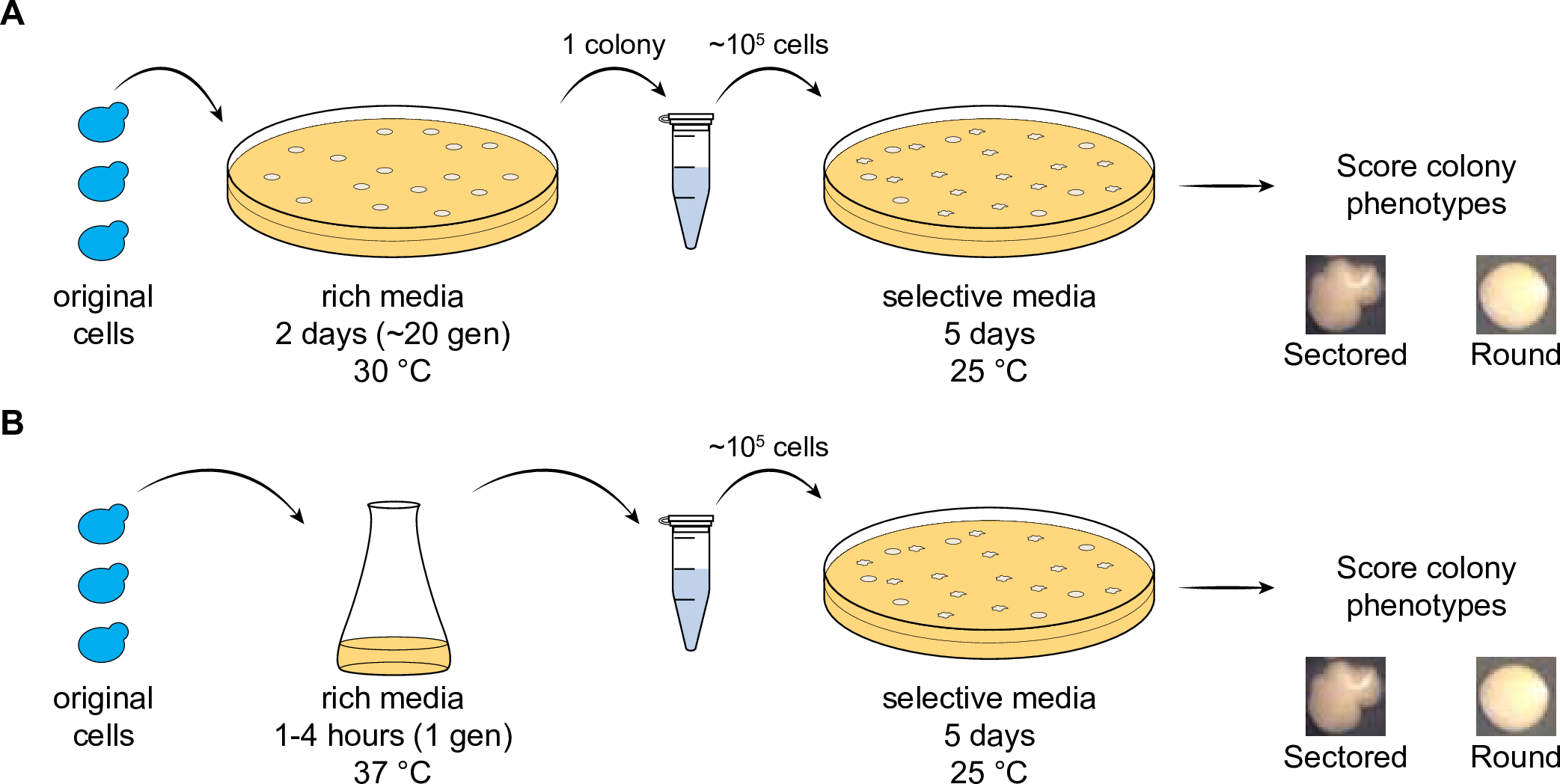

Supplement: S1 Fig — (A) Cells are plated to rich media for 2 days at 30°C (semi-restrictive temperature for cdc13F684S), then individual colonies are plated to canavanine-containing media for 5 days at 25°C to select for chromosomal rearrangements. Instability is scored by comparing the number of “sectored” or “round” colonies vs the number of cells originally plated. (B) Asynchronous cells are grown at 25°C then shifted to 37°C for 1-4h. Then ~105 cells are plated to canavanine-containing media for 5 days at 25°C to select for chromosomal rearrangements. Instability is scored by comparing the number of “sectored” or “round” colonies vs the number of cells originally plated. (TIF) [file pgen.1008733.s001.tif]

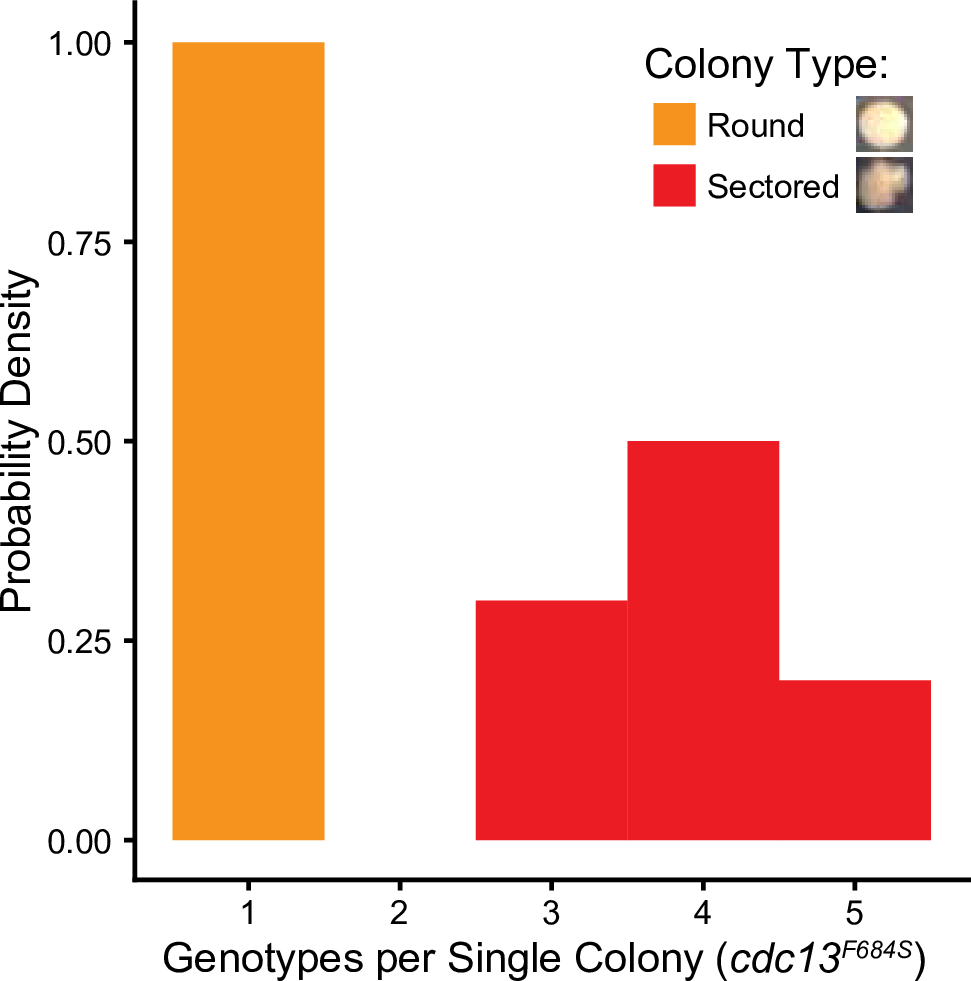

Supplement: S2 Fig — (A) Histogram for the number of genotypes in sectored (red) and round (orange) colonies, n = 20 (n = 10 colonies each for sectored and round; experiment done at 30°C). (TIF) [file pgen.1008733.s002.tif]

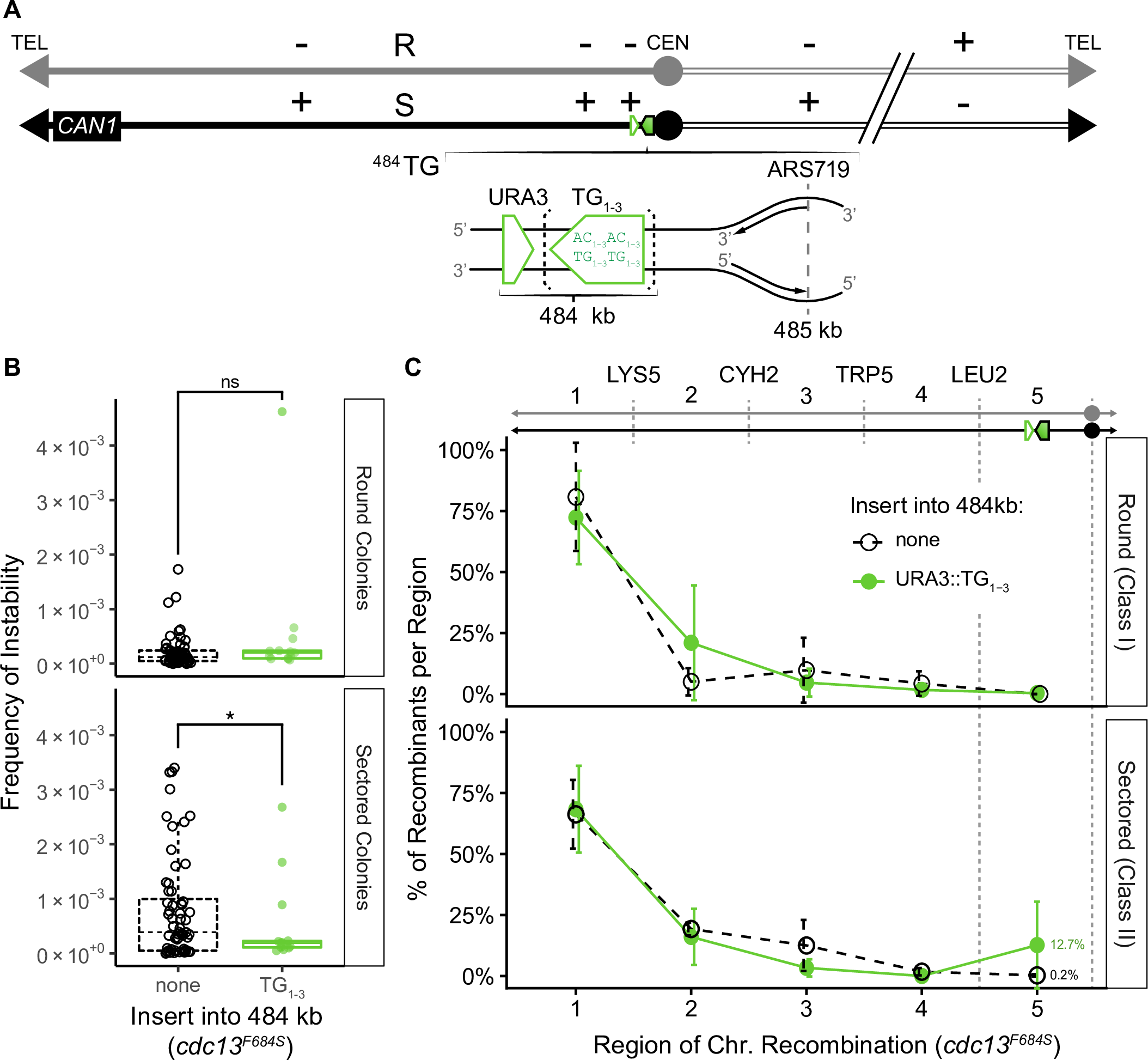

Supplement: S3 Fig — (A) Diagram for the TG repeat (green box) is insertion in the 484 kb locus. (B) Frequency of round (top) and sectored colonies (bottom) from an unmodified cdc13F684S and cdc13F684S 484TG1-3 are not altered. The median is shown (n > 12; * < 0.01; Mann-Whitney U). (C) Distribution of genotypes from round (Class I recombinants) and sectored colonies (Class II recombinants) from cdc13F684S with no insert or with the TG1-3 insert. The average percentage and standard deviation for 3 independent experiments are shown. (TIF) [file pgen.1008733.s003.tif]

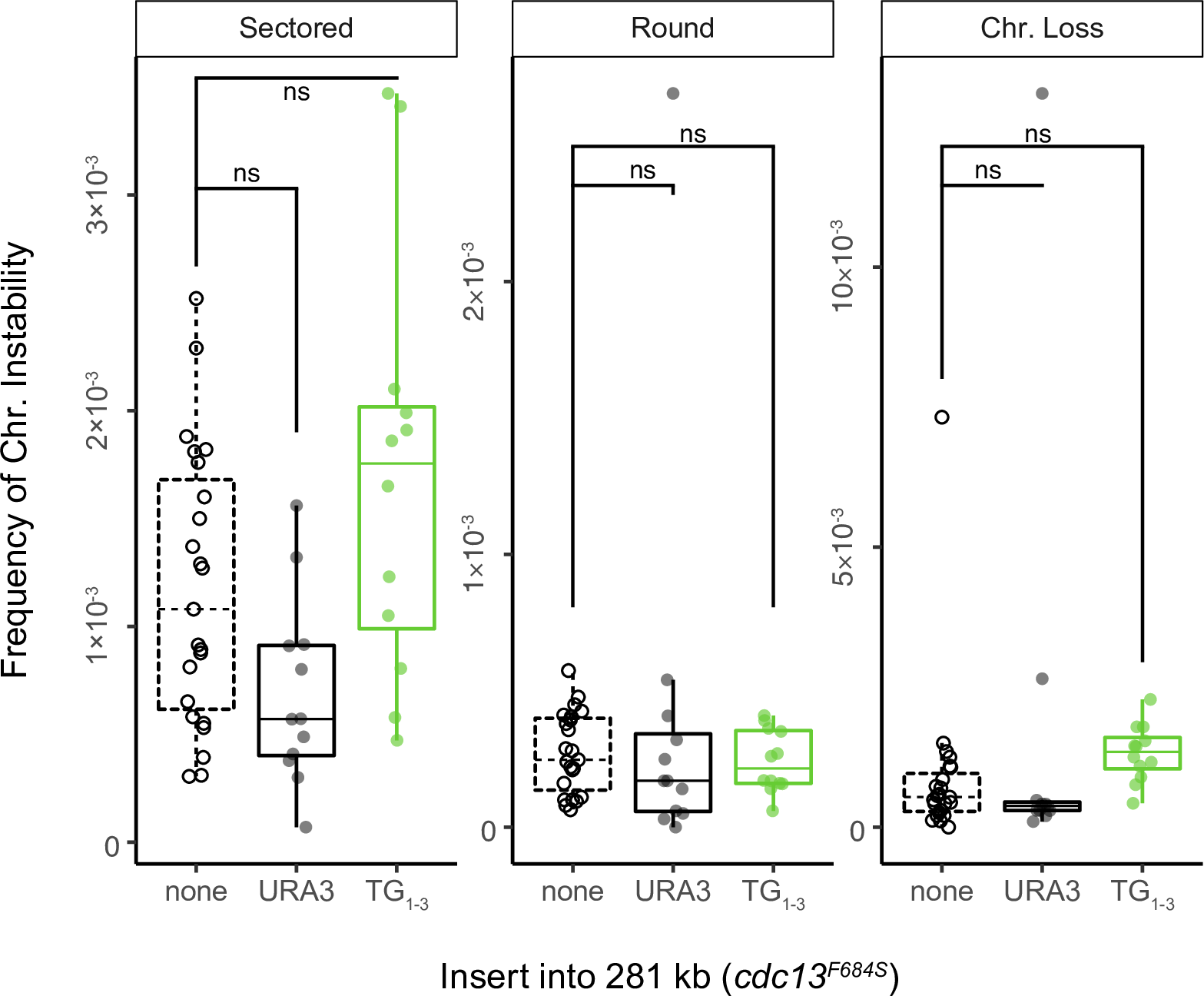

Supplement: S4 Fig — (A) Frequency of instability for sectored, round, and chr. loss colonies from an unmodified cdc13F684S, cdc13F684S URA3, and cdc13F684S 281TG1-3. The median is shown (n > 12; * < 0.01; Mann-Whitney U; experiment done at 30°C). (TIF) [file pgen.1008733.s004.tif]

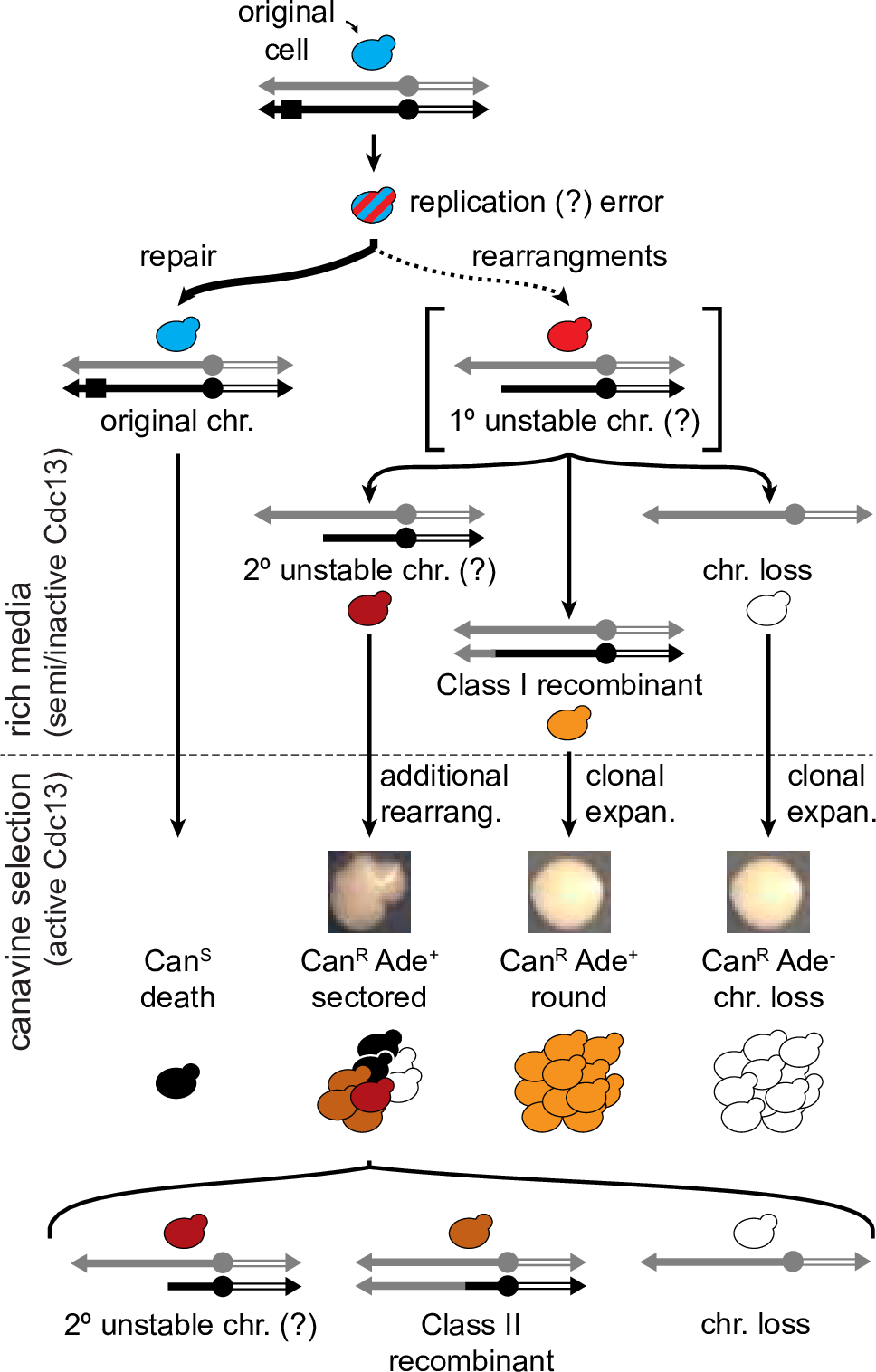

Supplement: S5 Fig — (A) Complete schematic of how diversity arises from one unstable chromosome. An unstable chromosome replicates, then various descendent chromosomes rearrange in subsequent cell divisions. Further explanation in text. (TIF) [file pgen.1008733.s005.tif]

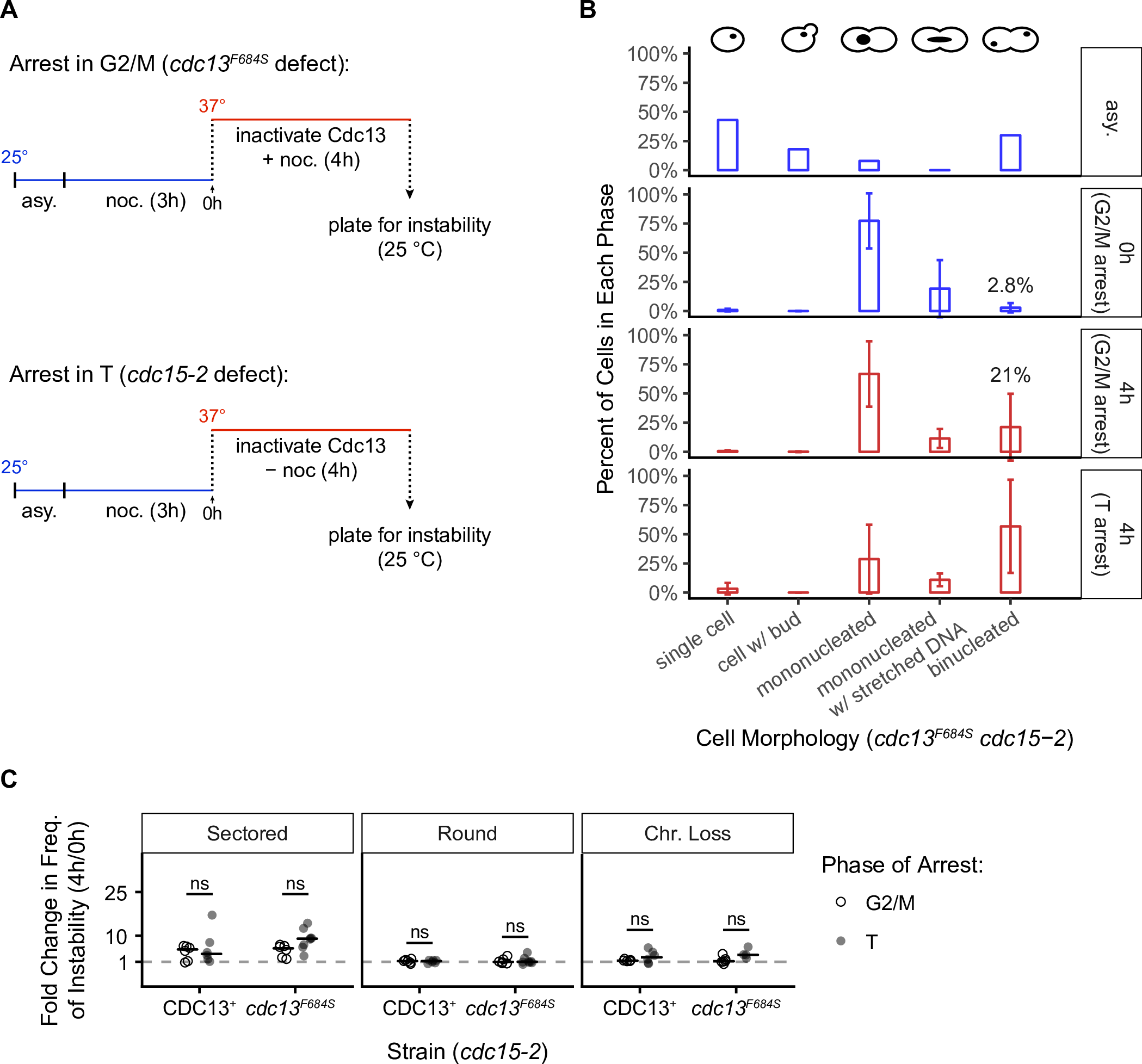

Supplement: S6 Fig — (A) Schematic for arresting cells in either G2/M or T phase. Cells were grown at 25°C until they reached mid-log, then were incubated with nocodazole (noc) for 3 hours. Cells were then washed (0h) and the culture was split. Both halves were incubated at 37°C for 4 hours, one with (4h + noc; top) and without (4h –noc; bottom). At asy, 4h + noc, and 4h –noc cells were collected for DAPI staining and the instability assay. (B) Nuclear morphology based on DAPI staining of chromosomes. The proportion of cdc13F684S cdc15-2 cells with each morphology for the different arresting protocols is shown. Blue: cells grown at 25°C; red: cells grown at 37°C. Mean and standard deviation from at least 6 independent experiments are shown. (C) Fold change in the frequency of sectored, round, and chr. loss colonies (4h/0h) from cdc15-2 CDC13+ and cdc13F684S cdc15-2 with (n = 6; 6) and without (n = 6; 8) additional nocodazole added after the initial noc arrest. The fold change between 4h and 0h remained low despite the arresting conditions (* < 0.05, Mann-Whitney U). (TIF) [file pgen.1008733.s006.tif]

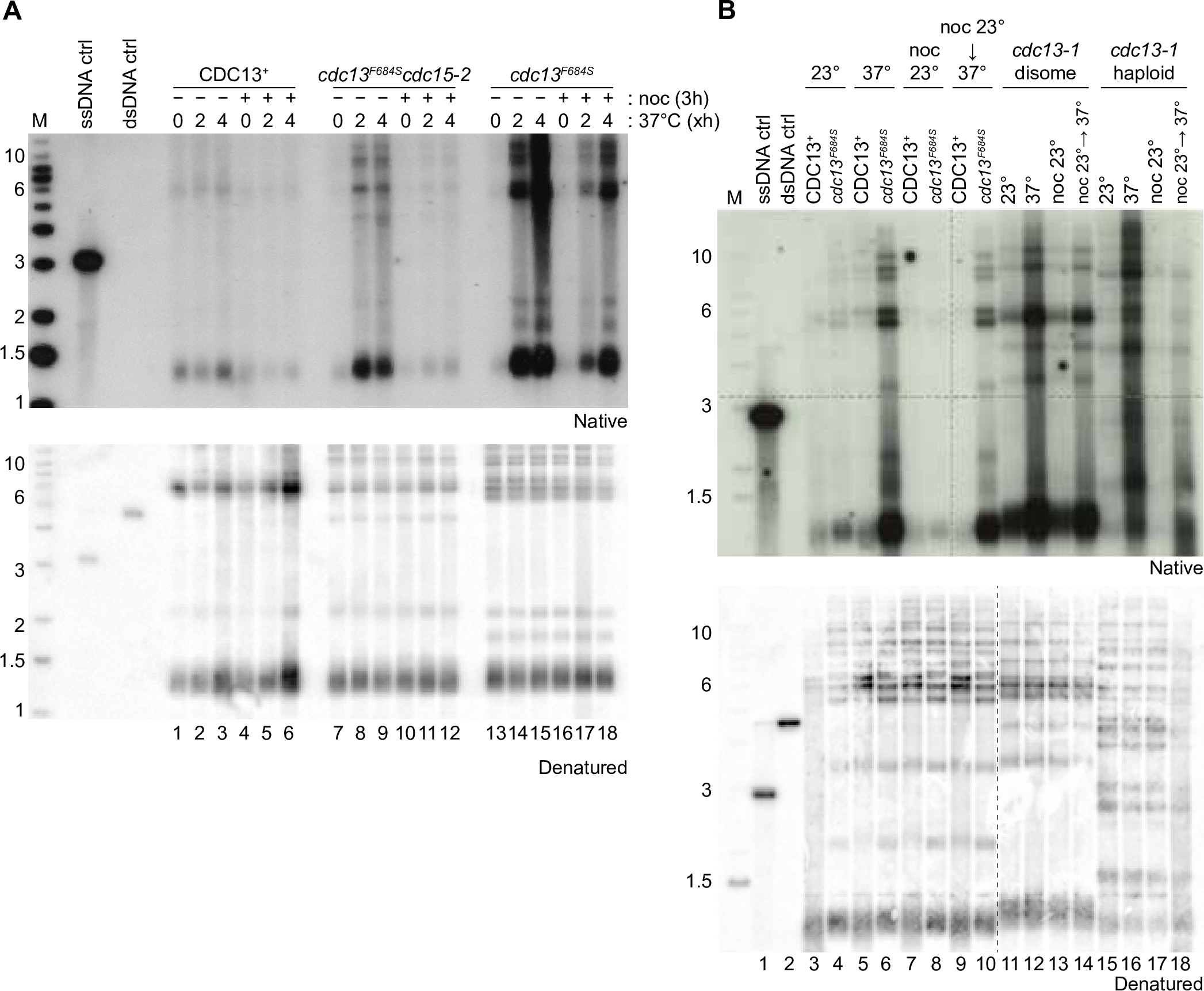

Supplement: S7 Fig — (A) Uncropped image of the non-denaturing in-gel hybridization in Fig 4E. Note that ssDNA is generated in cdc13F684S after nocodazole arrest at 25°C and release into 37°C (lanes 13–18; not depicted in Fig 4E). (B) Additional non-denaturing in-gel hybridization using a CA oligonucleotide probe with XhoI-digested DNA from the indicated samples (top). To control DNA input, the gel was denatured and transferred to a nitrocellulose membrane and hybridized with a telomeric probe (bottom). Cells were grown in the indicated conditions for 3 hours. (TIF) [file pgen.1008733.s007.tif]

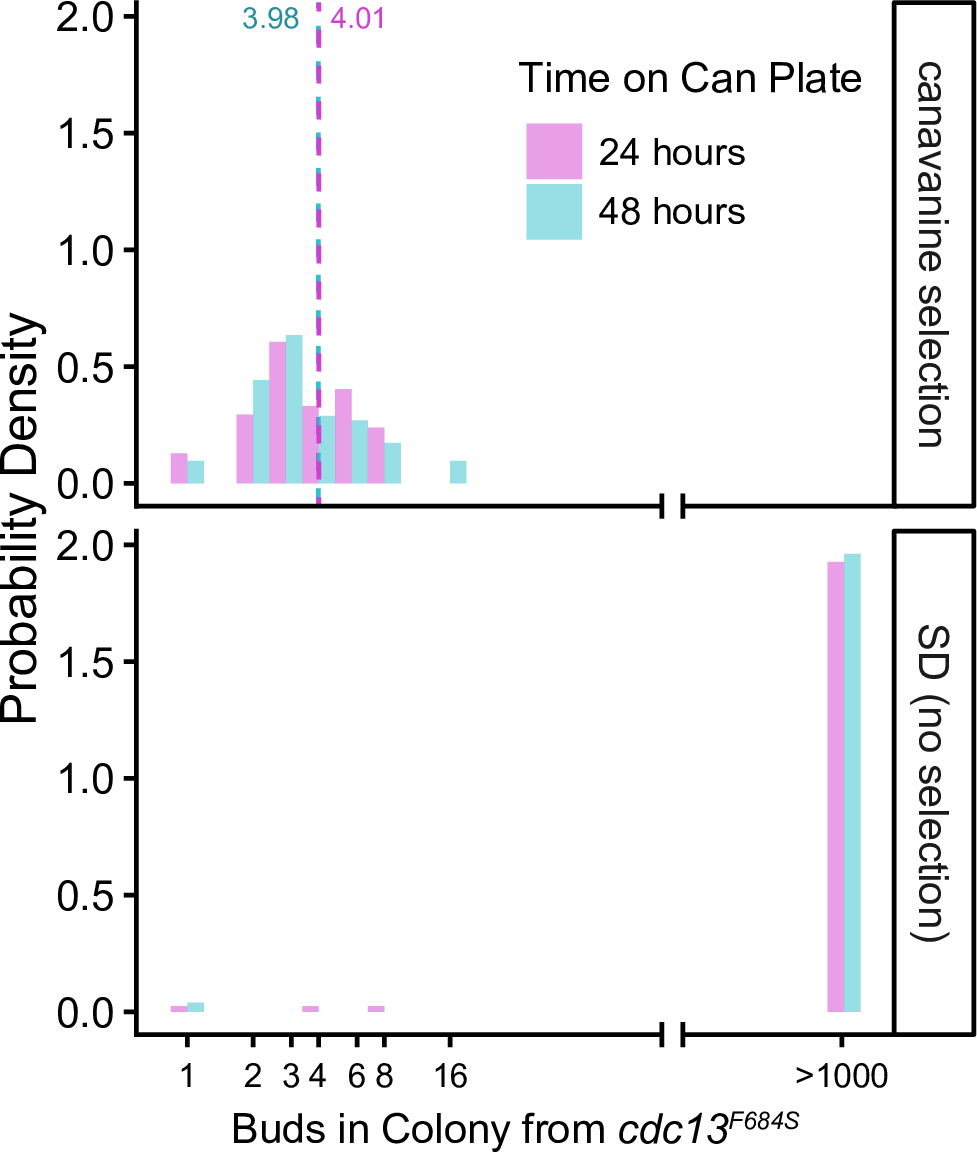

Supplement: S8 Fig — Density curve for the colony size of cdc13F684S (t0 cells from Fig 3A top grown on plates with (top) or without canavanine (bottom). Colony size was scored at 24h (purple) and 48h (blue). Dashed red line: the average number of buds per colony for cells grown with canavanine (mean = 4.01 buds). Experiment done at the permissive temperature (25°C). (TIF) [file pgen.1008733.s008.tif]

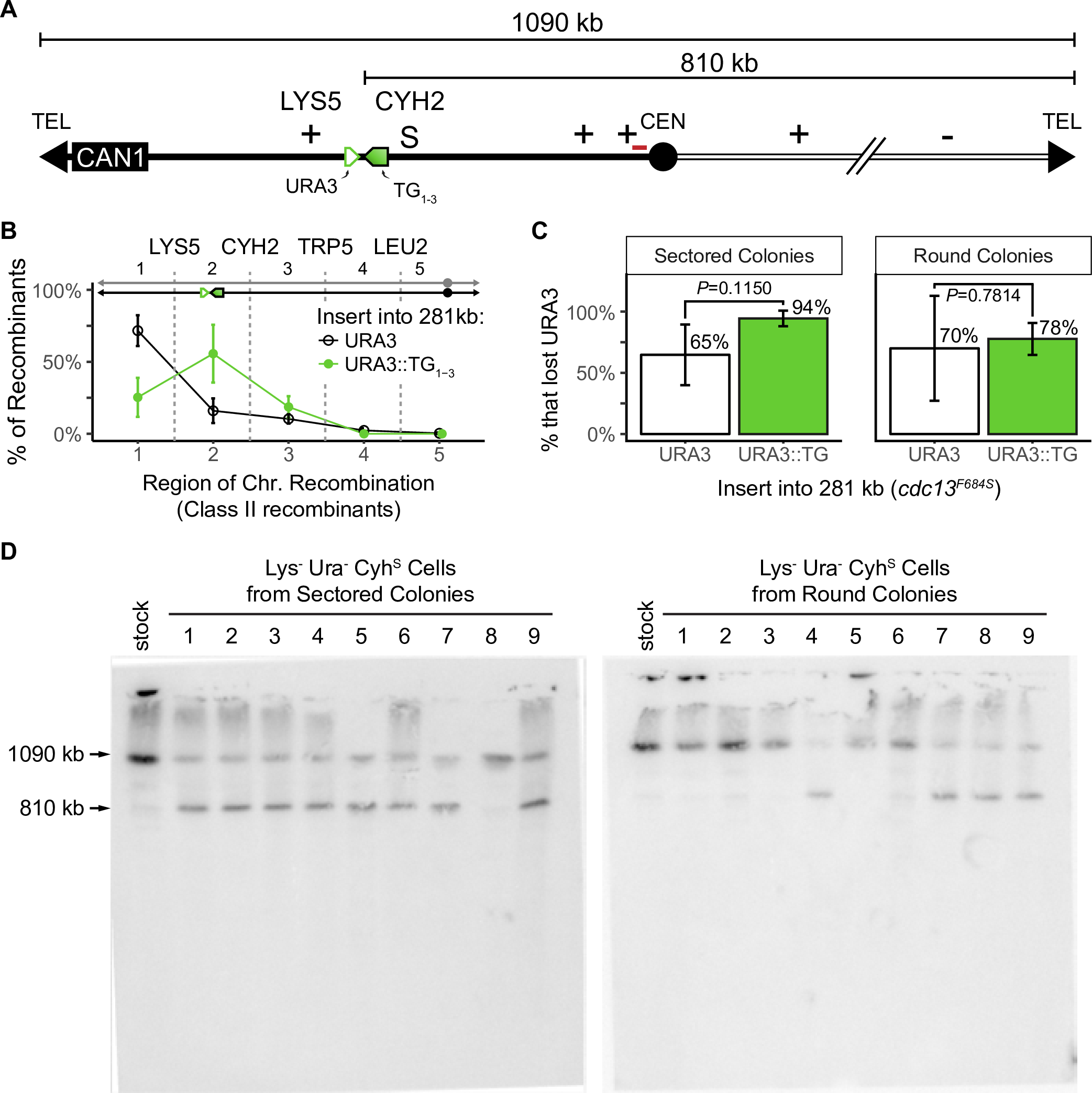

Supplement: S9 Fig — (A) The 281URA3 or 281TG repeat (green box) inserted between LYS5 and CYH2 as in Fig 2B. Red line indicates the probe binding site. (B) Distribution of Class II recombinants from sectored colonies from cdc13F684S with URA3 or URA3::TG1-3 inserted into the 281 kb locus. The average percentage and standard deviation for 3 independent experiments are shown. (C) Proportion of Lys- CyhS cells that have additionally lost URA3 from sectored and round colonies. P values were calculated with a t test. (D) Southern blot of pulse-field gels from Lys- Ura- CyhS cells from sectored or round colonies. A Chr VII centromere-linked probe was used to label chromosome VII. The upper band corresponds to the normal Chr VII size (1090 kb) while the lower band corresponds to a truncation at 281 kb (810 kb; localizes with Chr II, 813 kb). (TIF) [file pgen.1008733.s009.tif]

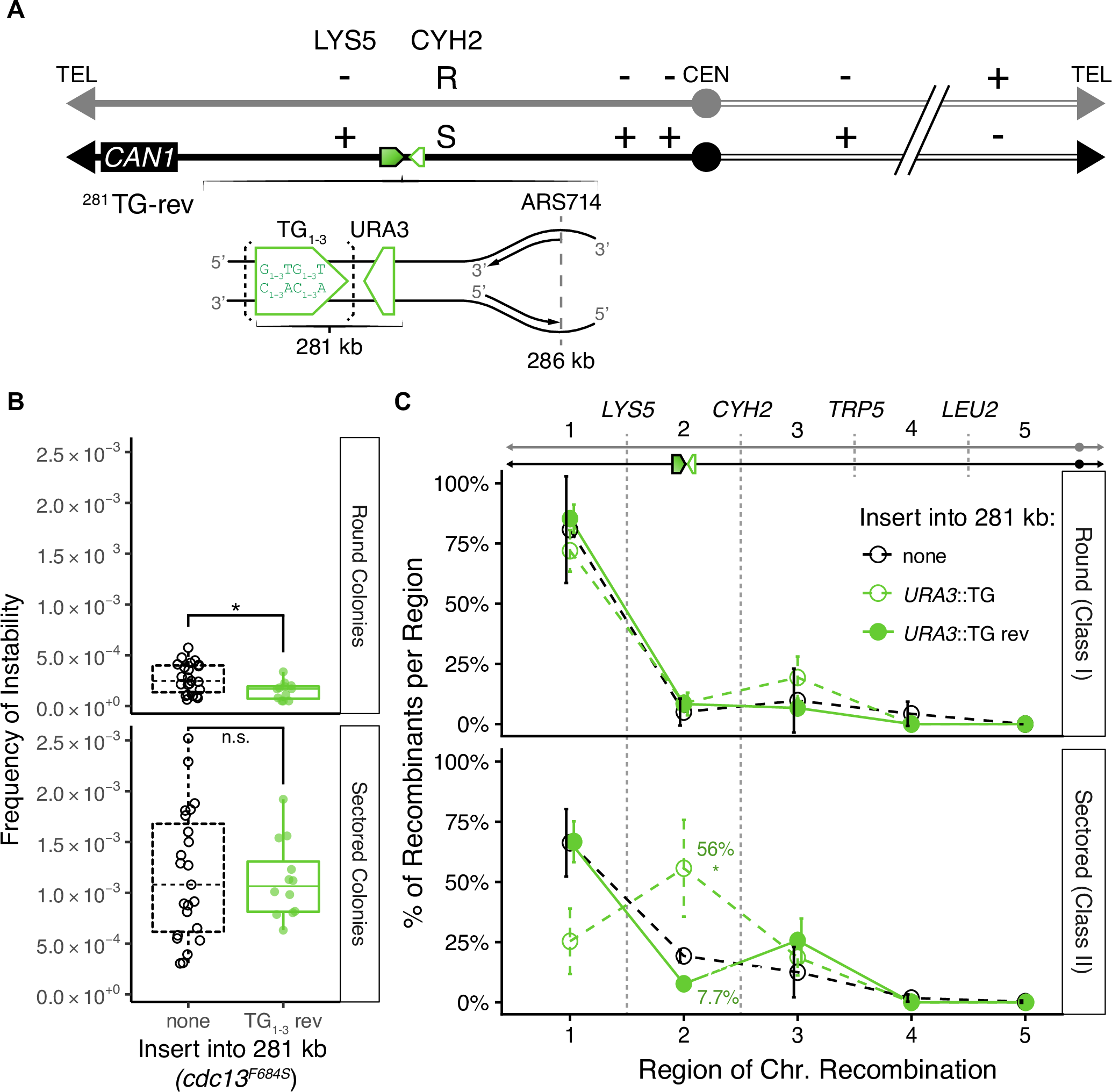

Supplement: S10 Fig — (A) The TG repeat (green box) in the 281 kb in the reversed orientation (TG-rich strand acting as the template for leading strand replication). (B) Frequency of round (top) and sectored colonies (bottom) from an unmodified cdc13F684S (white) and cdc13F684S 281TG1-3-rev (green) are not altered (n > 12; * < 0.01; Mann-Whitney U). (C) Distribution of genotypes from round (Class I recombinants) and sectored colonies (Class II recombinants) from cdc13F684S with no insert, with 281TG1-3, or with 281TG1-3-rev. The average percentage and standard deviation for 3 independent experiments are shown (P = 0.01; one sample t test). (TIF) [file pgen.1008733.s010.tif]
