# Supplementary material for: Loss of Cdc13 causes genome instability by a deficiency in replication-dependent telomere capping
Source: PLoS Genet. 2020 Apr 14;16(4):e1008733. doi: 10.1371/journal.pgen.1008733 (PMC7205313; doi:10.1371/journal.pgen.1008733)
Supplement: S1 Table — Median values and IQR, in []s, are reported. In bold, statistically significant (P < 0.01; Mann-Whitney U) fold change between single mutants and Wild Type (CDC13+) or between cdc13F684S mutX and cdc13F684S Normalized median frequencies are reported in ()s (normalized to CDC13+). (DOCX) [file pgen.1008733.s011.docx]

**S1 Table. Median frequencies of chromosome instability in additional *cdc13^F684S^* mutants at 30 °C**

|  | **Strain** | **Sectored (×10^-5^)** | **Round (×10^-5^)** | **Chr Loss (×10^-5^)** |
| --- | --- | --- | --- | --- |
|  | Wild Type (CDC13^+^) | 2.9 [3.4] (1.0) | 7.1 [4.5] (1.0) | 12 [38] (1.0) |
|  | *cdc13^F684S^* | **88 [120] (30)** | **20 [20] (2.8)** | **73 [93] (6.1)** |
| Telomerase-regulators | *pif1-m2* | **33 [32] (11)** | 6.9 [2.3] (0.97) | **140 [32] (12)** |
|  | *hrq1Δ* | 3.8 [2.2] (1.3) | 7.0 [4.9] (0.99) | 15 [14] (1.3) |
|  | *pif1-m2 hrq1Δ* | **62 [29] (21)** | 6.1 [6.0] (0.86) | **230 [160] (19)** |
|  | *cdc13^F684S^ pif1-m2* | **30 [41] (10)** | **5.9 [6.5] (0.83)** | **20 [37] (1.7)** |
|  | *cdc13^F684S^ hrq1Δ* | 160 [86] (55) | 18 [35] (2.5) | 60 [57] (5.0) |
|  | *cdc13^F684S^ pif1-m2 hrq1Δ* | **8.5 [19] (2.9)** | **5.5 [3.6] (0.77)** | **19 [25] (1.6)** |
| Helicase | *rrm3Δ* | 7.9 [4.2] (2.7) | **1.0 [0.50] (0.14)** | 46 [140] (3.8) |
|  | *cdc13^F684S^ rrm3Δ* | 120 [70] (41) | **6.5 [11] (0.92)** | 60 [44] (5.0) |
| PRR | *rad18Δ* | **200 [120] (69)** | **55 [8.3] (7.7)** | **460 [410] (38)** |
|  | *cdc13^F684S^ rad18Δ* | **230 [51] (79)** | **150 [120] (21)** | 570 [350] (48) |
| DNA damage checkpoint | *rad17Δ* | **900 [370] (310)** | **55 [120] (7.7)** | **490 [190] (41)** |
|  | *cdc13^F684S^ rad17Δ* | **520 [660] (180)** | **310 [770] (44)** | **340 [350] (28)** |
|  | *tel1Δ* | **60 [21] (21)** | **54 [18] (7.6)** | **93 [38] (7.8)** |
|  | *cdc13^F684S^ tel1Δ* | 180 [66] (62) | **140 [69] (20)** | 190 [70] (16) |
|  | *xrs2Δ* | **450 [410] (160)** | 11 [28] (1.5) | **2000 [1600] (170)** |
|  | *cdc13^F684S^ xrs2Δ* | **560 [220] (190)** | **3.0 [2.1] (0.42)** | **1100 [660] (92)** |
| Median values and IQR, in []s, are reported.  In bold, statistically significant (*P* < 0.01; Mann-Whitney U) fold change between single mutants and Wild Type (CDC13^+^) or between *cdc13^F684S^ mutX* and *cdc13^F684S^*  Normalized median frequencies are reported in ()s (normalized to CDC13^+^). | | | | |
