# Supplementary material for: Loss of Cdc13 causes genome instability by a deficiency in replication-dependent telomere capping
Source: PLoS Genet. 2020 Apr 14;16(4):e1008733. doi: 10.1371/journal.pgen.1008733 (PMC7205313; doi:10.1371/journal.pgen.1008733)
Supplement: S2 Table — a All strains are disomic for Chr VII and are derivatives of TY200 MATα +/hxk2::CAN1 lys5/+ cyhr/CYHS trp5/+ leu1/+ Centromere ade6/+ +/ade3, ura3-2. b The URA3 module, TG1-3 repeat, and/or selective markers were integrated into the CAN1 homologue of Chr VII. The insert locations are notated (kb from the left telomere of Chr VII). * Double mutants integrated into cdc13F684S with altered chromosomes V and VIII. (DOCX) [file pgen.1008733.s012.docx]

**S2 Table. *Saccharomyces cerevisiae* strains used in this study**

| Strain | Genotype^a,b,*^ | Source |
| --- | --- | --- |
| TY200 | CDC13^+^ (Wild Type) | Admire et al 2006 |
| TY629 | *cdc13-F684S::ura3* | This study |
| TY630 | *cdc13-Y556A,Y558A::ura3* | This study |
| TY631 | *stn1-T223A,S250A::ura3* | This study |
| TY632 | *cdc13-F684S::ura3 stn1-T223A,S250A::ura3* | This study |
| TY712 | *cdc13-F684S::ura3 stn1-T223A,S250A::ura3 75::HPH* | This study |
| TY588 | *CDC13^+^ 403::RA::nat1MX::RU 535::A3::KanMX4* | Beyer & Weinert 2016 |
| TY633 | *cdc13-F684S::ura3 403::RA::nat1MX::RU 535::A3::KanMX4** | This study |
| TY634 | *cdc15-2::ura3* | This study |
| TY635 | *cdc13-F684S::ura3 cdc15-2::ura3* | This study |
| TY714 | *sir2Δ::KanMX4* | This study |
| TY713 | *cdc13-F684S::ura3 sir2Δ::KanMX4* | This study |
| TY436 | *rad52Δ::URA3* | Paek et al. 2009 |
| TY636 | *cdc13-F684S::ura3 rad52Δ::KanMX4** | This study |
| TY447 | *lig4Δ::KanMX4* | Paek et al. 2009 |
| TY637 | *cdc13-F684S::ura3 lig4Δ::KanMX4** | This study |
| TY516 | *exo1Δ::URA3* | Kaochar et al. 2010 |
| TY638 | *cdc13-F684S::ura3 exo1Δ::KanMX4** | This study |
| TY639 | *sae2Δ::URA3/KanMX4* | This study |
| TY640 | *cdc13-F684S::ura3 sae2Δ::URA3/KanMX4** | This study |
| TY900 | *sae2Δ::URA3/KanMX4 rad9:HPH* | This study |
| TY641 | *pif1-m2::ura3* | This study |
| TY642 | *cdc13-F684S::ura3 pif1-m2::ura3** | This study |
| TY643 | *hrq1Δ::KanMX* | This study |
| TY644 | *cdc13-F684S::ura3 hrq1Δ::KanMX4** | This study |
| TY645 | *pif1-m2::ura3 hrq1Δ::KanMX* | This study |
| TY646 | *cdc13-F684S::ura3 pif1-m2::ura3 hrq1Δ::KanMX4** | This study |
| TY591 | *rrm3Δ::KanMX4* | Beyer & Weinert 2016 |
| TY647 | *cdc13-F684S::ura3 rrm3Δ::KanMX4** | This study |
| TY440 | *rad18::KanMX4* | Paek et al. 2009 |
| TY648 | *cdc13-F684S::ura3 rad18::KanMX4** | This study |
| TY660 | *rfa-t33::ura3* | This study |
| TY661 | *cdc13-F684S::ura3 rfa-t33::ura3* | This study |
| TY206 | *rad9::ura3* | Admire et al. 2006 |
| TY649 | *cdc13-F684S::ura3 rad9::ura3* | This study |
| TY216 | *rad17::hisGura3* | Admire et al. 2006 |
| TY650 | *cdc13-F684S::ura3 rad17::KanMX4** | This study |
| TY500 | *tel1Δ::HPH* | Kaochar et al. 2010 |
| TY651 | *cdc13-F684S::ura3 tel1Δ::HPH** | This study |
| TY522 | *xrs2Δ::KanMX4* | Kaochar et al. 2010 |
| TY652 | *cdc13-F684S::ura3 xrs2Δ::KanMX4** | This study |
| TY653 | *cdc13-F684S::ura3 281::TG_1-3_::URA3* | This study |
| TY654 | *cdc13-F684S::ura3 281::URA3* | This study |
| TY655 | *cdc13-F684S::ura3 rad52Δ::KanMX4 281::TG_1-3_::URA3* | This study |
| TY656 | *cdc13-F684S::ura3 281::TG_1-3_-rev::URA3* | This study |
| TY657 | *cdc13-F684S::ura3 287::TG_1-3_::URA3** | This study |
| TY629 | *cdc13-F684S::ura3 484::TG_1-3_::URA3** | This study |
| TY630 | *cdc13-F684S::ura3 122::KanMX4 281::TG_1-3_::URA3* | This study |
| TY631 | *cdc13-F684S::ura3 122::KanMX4 281::URA3* | This study |
| ^a^ All strains are disomic for Chr VII and are derivatives of TY200 MATα +/hxk2::CAN1 lys5/+ cyh^r^/CYH^S^ trp5/+ leu1/+ Centromere ade6/+ +/ade3, ura3-2.  ^b^ The URA3 module, TG_1-3_ repeat, and/or selective markers were integrated into the *CAN1* homologue of Chr VII. The insert locations are notated (kb from the left telomere of Chr VII).  * Double mutants integrated into *cdc13-F684S* with altered chromosomes V and VIII. | | |
