# Supplementary material for: Loss of Cdc13 causes genome instability by a deficiency in replication-dependent telomere capping
Source: PLoS Genet. 2020 Apr 14;16(4):e1008733. doi: 10.1371/journal.pgen.1008733 (PMC7205313; doi:10.1371/journal.pgen.1008733)
Supplement: Attachment — Submitted filename: Response to reviewers.docx [file pgen.1008733.s014.docx]

Revised submission to PLOS Genetics (PGENETICS-D-19-01631) [EMID:1f76b2017f251c5a]

Dear Editors of PLOS Genetics

We hereby resubmit our manuscript entitled   'Loss of Cdc13 causes genome instability by a deficiency in replication-dependent telomere capping' .

We thank you heartily for allowing us more time, twice over, to complete revisions.

We address all reviewer’s comments. We completed one key experiment involving Sae2, with a twist in its completion, as described in the text and in our response to reviewer’s comments.

Please let us know if there are any issues we have left unresolved.

Regards

Ted Weinert

Professor Molecular and Cellular Biology

**Comments to the Authors:
Please note here if the review is uploaded as an attachment.**

Reviewer #1: The authors investigated the role of Cdc13 in maintaining genome stability. By using the temperature sensitive cdc13-F684S, they show that the passage through S phase is necessary to generate chromosome instability (recombinants, chromosome truncations, dicentrics, and/or loss) in cdc13-F684S mutant. Generation of unstable chromosomes requires Exo1, but not homologous recombination and non-homologous end joining. The authors propose a model where Cdc13 dysfunction impairs semi-conservative replication at the telomere that leads to ssDNA and unstable chromosomes.

The manuscript is interesting and the experiments are well done. The major finding that instability in cdc13 mutants is replication dependent is novel and important.

I have some suggestions that might help to improve the understanding of the manuscript:
1. Lines 157-159: the interpretation of the synergistic increase in cdc13 stn1 double mutant is difficult to understand. As Cdc13 and Stn1 interact, it could simply be that each single mutation does not fully impair Cdc13 and Stn1 function but, when they are both present in the same cells, this lead to a more severe defect of CST function that could explain the synergistic effect.

We agree with this interpretation and rewrote the stn1 conclusion to make this more clear (lines 177-180).

2. Lines 240-248: Since telomerase dependency was not tested, I was asking whether it is necessary to distinguish lagging strand synthesis after telomerase or independent of telomerase…

We agree that we cannot distinction between Cdc13’s role in lagging-strand fill-in dependent or independent of telomerase, and replaced “lagging strand synthesis after telomerase extension” with “C-strand fill-in” (line 268, 299).

3. Page 252: delete the sentence “A method…” or explain why it is not possible to inactivate Cdc13 only in S phase (what about releasing cells from a G1 arrest at 37°C?).

As suggested we deleted this sentence.

4. Page 329-345: It is quite clear that the lack of Sae2 suppresses instability in cdc13 mutant, but the effect on rfa1-t33 is difficult to understand. The lack of Sae2 impairs hairpin cleavage but it also increases Rad9 binding at DSBs. Since the lack of Rad9 increases instability in cdc13 mutants, it could also be that the lack of Sae2 suppresses instability by increasing Rad9 binding. This could be tested in cdc13 rad9 sae2 triple mutant to see whether suppression depends on Rad9.

We tried to make the triple, by transforming a *cdc13 sae2* with rad9:HYG. Shockingly, we seemed to have recovered triple mutants with very peculiar phenotypes: cells no longer arrested at 36C, and they generated a very low frequency of CanR colonies, even at 25C, the permissive temperature for *cdc13^F684S^*, where the triple mutant should behave like *sae2 rad9*. Something puzzling (duplicated *CAN1*?) and interesting (failure to arrest) is going on that requires further study. Nevertheless, our *sae2 rad9* results are conclusive that, at least in a CDC13+ cell; *sae2* is not suppressing *rad9* instability (Table 2).

In any case, the authors propose that “it is unlikely that the hairpin mechanism is responsible for chromosome instability”. However, the model includes hairpin cleavage by Sae2. Furthermore, at line 424 they “suggest a role for cleavage of hairpins…”. This is confusing…

We reworded this section to make it clear that the model proposed by Deng et al 2015 was unlikely to cause our chromosome instability. We wrote it to say: “In conclusion, the mechanism described by Deng et al is not responsible for the formation of unstable chromosomes in our system.” (lines 386-387). We also deemphasized the possible role of hairpins in our model.

Reviewer #2: Langston et al investigate the timing of action of a cdc13ts allele and the types of chromosome instability that are found as a consequence of loss of cdc13 function under a variety of different conditions that depend on the presence of active Cdc13 is S and G2/M phases or only in the G2/M phase. The authors find that the S phase activity is required for the cdc13ts defects and form two class of recombinants as well as chromosome loss events. The authors demonstrate through dependence on Exo1 and through identification of single-stranded telomeric DNA in G2/M that single-stranded regions are critical for the formation of recombinants. The recombinants are favored at telomere proximal regions and are not simply due to the presence of TG repeats that, when present at distal sites, do not change the products that are formed with the exception of a truncation at 482 kb.

These data are highly important as they demonstrate the need for DNA replication for Cdc13 activity that prevents recombination and chromosome loss. The work is thorough and, for the most part, experiments are well conceived. The quality of the data is, in general, very high. The paper could have been written more clearly and there are grammatical mistakes throughout. More importantly, there are explanations and interpretations that are oversimplified or omitted that require clarification and stipulation. The specific major and minor criticisms are enumerated below.

Major criticisms:
1. There are several stipulations regarding the compared shifts into various media.
These are based on several technical limitations. First, cells that shift the cdc13 ts after growth to nocodazole (Figure 4) may have already passed through part of the G2 phase. The possibility for some activity in the G2 phase still remains. This finding needs to be clearly stipulated. Furthermore, the authors do not discuss whether the cdc13ts arrests before or after the nocodazole point. In fact, it is impossible to ascertain since after the shift to 37°C nocodazole is again added. The fact that the phenotype depends on growth at 37°C, however, argues for an effect of the mutant so it probably acts at or near the nocodazole point.

Yes, formally Cdc13 could act in G2/M before the noc arrest. The experiment, though, is asking a simpler question: does Cdc13 cap the chromosome post replication. If the chromosomes had been unstable, the answer would have been yes, Cdc13 caps the chromosome. The chromosomes were stable, however, raising the question what does Cdc13 do and when.

2. The comparison between the shifting experiments are not always appropriate. In Figure 3, a shift of asynchronous cells to 37, allowing both S and G2/M arrest, is compared to cells synchronized and maintained with HU, a DNA synthesis inhibitor, followed by shift to 37C. The former yields less of an increase in instability than the latter, leading to the conclusion that incubation with HU before S phase participates the subsequent S phase activity of Cdc13. What is important is that both of these schemes that lead to S phase involvement. A better comparison would be arrest with alpha factor before wash-out before shift-up in growth that would also be presumably S phase to continue until G2/M arrest. Arrest of cells in HU could simply increase the frequency of recombinants by limiting the cells analyzed to arrested cells rather than to cells at any cell cycle stage. This lack of parallel approach does not take away from the interest in HU, although it is not central to the paper. The experimental protocol is simply not parallel. The comparison is also not appropriate in Figure 4 were asynchronous cells are compared to nocodazole arrested cells before shift-up. Again, the comparison between asynchronous cells and synchronized cells are not parallel. Lines 236-238 are overstated.

We did not use alpha factor for a technical reason, and liked the enhanced phenotype of using HU. We have not yet solved the technical problem.

The enhancement by HU is not likely because more cells experience a cdc13-defect from HU-arrested cells than from asynchronous cells. So, by our reckoning, about the same number of cells in both protocols transit an S phase without Cdc13, +/- 25%.

For example, from HU-arrested, say 100% of the cells experience S phase without Cdc13 after shift-up. How many cells from an asynchronous culture experience an S phase without Cdc13? From an asynchronous culture, say 66% of the cells are before S phase and after the previous G2/M; these cells will experience S phase without Cdc13. And those cells in S phase to G2/M in an asynchronous culture? Most will experience S phase without some Cdc13, as cells that do not incur enough damage will actually cycle thru to the next S phase (execution point of *cdc13-1* is about 0.4, middle of S phase).

We toned down the conclusion from lines 236-238 (now lines 263-265) to now say “This paradigm should allow us to narrow down the timeframe for when Cdc13 must be active, in S phase and/or in G2 phase, to preserve chromosome stability”

3. The characteristics of the ts allele is that it is degraded very rapidly at the restrictive temperature. This indicates that any events after shifting down to 25°C does not involve immediate renaturation, but rather re-synthesis of the protein. This should be noted, although it does not affect the interpretation of the data, it may influence the timing of recovery. Hence on line213, “We then reactivated the protein” should probably be replaced by “We then resynthesized the protein”.

We agree and changed the wording from “reactivated” to “resynthesized” (lines 119, 238, 294, 502, 507).

4. The authors should draw or explain Class I and Class II recombinants after they are first mentioned.

We added a brief description of Class 1 and II recombinants when they are first described, Fig 1Bii (lines 127-130). We subsequently address Class I and II more extensively in a later section. Finally, we remove the use of “Class 1” recombinant to refer to round colonies (“round” is the phenotype for Class 1 recombinants) to avoid unnecessary confusion.

5. How are the plots of the expectation of random recombination determined?

The probability of random recombination is based on the length of the region divided by the total length of the arm, the explanation was added to the Fig 2A legend.

6. Line 159: How do the authors explain the lack of stn1 mutant phenotype in chromosome instability?

We reason that the stn1 mutation alone does not fully impair Cdc13 and Stn1 function, we reworded the conclusion in the text to make this more clear (as suggested by Reviewer 1, lines 177-180).

7. Lines 239-248. In the logical argument for two among the four possible mechanisms of Cdc13 activity, telomerase activity is eliminated and, logically, so is lagging strand activity associated after telomerase extension. However, please consider the possibility (textually) of lagging strand synthesis facilitation in the absence of telomerase extension.

We include the possibility of Cdc13’s role in C-strand fill in after the replication fork on line 268 since we can’t distinguish between Cdc13’s role at the replication fork itself and its role filling in the C-strand as the overhang on the leading telomere matures.

8. Line 531-533: Please describe the alterations. The vague description is not acceptable for the acceptance of the corresponding data.

We added in more details about what was altered (line 578). We found by pulse-field gels that Chr VIII and V have altered sizes, and therefore underwent some translocation event. Neither copy of Chr VII was affected.

9. Tables 1 and 2. The meaning of the bold indication of significance is incomplete. Why are chromosome loss events not listed as significant?

The bold indications of significance in Tables 1 and 2 are complete, and significant events are bolded for chromosome loss. In the case of the tables, significance refers to either comparing *cdc13* to wild type, or the *cdc13-F684S mutX* to *cdc13-F684S*. For example, while *cdc13-F684S sae2*’s loss (89x10-5) is significantly increased compared to WT, it is not significantly different from *cdc13-F684S* (73x10-5), and is therefore not bolded. We altered the Table legends to make this more clear.

10. Why do statistical tests vary between t tests and Rank Sum in different experiments? Please explain.

We used Mann-Whitney U (rank sum) for the instability assays because sectoring, round, and chromosome loss were spontaneous events and we did not want to assume a Gaussian distribution. We used t tests when comparing regions of rearrangement because the Gaussian distribution assumption was appropriate.

11. Figure Legends have multiple errors and omissions that confuse the reading of the Figures:
a. Line 899: replace with “heterozygous markers”.

We agree and made the change.

b. Line 901: What do” replicas” refer to?

“Replicas” referred to the chromosomes descendent from the original unstable chromosome. The legend has been updated to make this more clear.

c. Asterisks are defined in Figure 1 Legend but are not present in Figure 1.

Asterisks were added in.

d. The temperature of growth in each experiment must be noted in the Figure Legends for interpretability.

Unless stated otherwise, experiments were done at 30 °C, legends have been updated to make this clear.

e. The cdc15-2 mutation must be introduced in the text associated with Figure 3, rather than Figure 4, since it is used in the former figure.

The *cdc13-F684S* *cdc15-2* mutation was only used for the Western blot in Fig 3B, and not anywhere else in the experiment describe in Fig 3, so we did not introduce the *cdc15-2* mutation in the text to avoid confusion. We updated the Fig 3B legend to explain this.

Minor criticisms: Textual recommendations
1. Line 66, Please define Cdc13/CST.

We clarified that we are referring to Cdc13 as a part of the CST complex (line 67).

2. Line 70, change “overhand” to “overhang”.

We corrected this typo.

3. Line 91, please give background on Sae2 function.

We removed Sae2 from the introduction since that result is more difficult to interpret without the *cdc13-F684S sae2 rad9* triple mutant. We include a more complete background of its function in lines 363-364 and 368.

4. Line 106, change “live” to “grow on canavanine-containing media”.

We agree and made this change.

5. Lines 112-114, the description is clumsy, please rewrite.

We redid this entire section to make the model in Fig 1B simpler. We explain the previously-studied 403 site (originally explain in lines 112-114), in a new paragraph (lines 131-138).

6. Throughout the manuscript, the authors compare or list phenotypes and the type of aberrant events in sentences creating confusion. For example, round Can R Ade+ with recombinant forms and Can R Ade- with chromosome loss events are intermixed Choose either the event or the phenotype in any given sentence.

We agree that this became confusing. We kept the marker description in the lines 122-123 when we describe the system we’re using, but we removed the reference to the markers from the rest of the Results. We also removed the use of “Class I” to refer to “round colonies” to not intermix the phenotype with the event.

7. Change all ‘loss’ events to ‘chromosome loss’ events.

We made this change.

8. Example: line 119: change to “unchanged cell dies on canavanine due loss of the dominant Can1S gene, form unstable…...and chromosome loss in CanRAde2- colonies. Another example in line 141 to 142.

See above

9. Delete “Two additional features of this model that”. It is redundant. “The unstable chromosomes give rise to both recombinant and chromosome loss. There are two class of recombinants. We theorize….

We agree that the former sentence structure was unwieldy and have made the suggested change.

10. Throughout capitalize CDC13 when mentioned as a gene.

We made the suggested change.

11. Line 131, Change “introduced “to “integrated”

We made the suggested change (line 147).

12. Line 139: change to “and plated onto selective media as an assay for chromosome instability.”

We changed the original “plated for instability” to “selective media to detect chromosome instability” (line 149).

13. Line 149: In the absence of evidence, change “many, if not all” to “multiple”.

We reworded this sentence to make it more clear that our conclusion is that the *cdc13*-dependent unstable chromosomes are not a Chr VII specific phenotype (lines 159).

14. Line 163: There is a general enthusiasm in the use of colons and “ yet it could” phrases in the text. In almost every case, a break between two sentences would be more appropriate

We read through and broke up long, unwieldy sentences as appropriate. We also reworked line 163 (now line 184-186) as suggested.

15. Line 167: the meaning of “linked to the site an error” is unclear. What kind of error?

Error here refers to the *cdc13-F684S*-dependent error in the DNA which causes the formation of the unstable chromosome. We simplified the sentence and clarified this in the text (lines 189-190).

16. Line 170: Eliminate “heavily” or give values.

We removed “heavily” (line 193).

17. Line 308: replace “phenotypic lag they persist” with “phenotypic lag that persists…”

This typo has been corrected.

18. Line 309-310: Remove question. It is unnecessary.

We removed the question (line 336).

19. E.g. Line 331: Deng et al should be followed by a reference in each case it is mentioned.

We agree and references to Deng et al were added when their conclusions were referenced (as opposed to relying on the numbered Deng *et al* citation in the text; line 382-387).

20. Lines 425-426: Please re-write extended sentence of phenotypes generated.

We removed the long list of phenotypes generated and simplified the sentence to focus on the main conclusion that the initial telomere-linked unstable chromosome is responsible for all the downstream rearrangements we see (lines 472-474).

21. Line 499: Define “various intervals”. Another way of looking at the very rare activities is as a selection for viability. Some of these events are very uncommon unless other frequent solutions are eliminated.

“Various intervals” refers to the different regions we can track with our heterozygous markers, we reworded the sentence to make this more clear (line 552-553). We agree that there are events that are more likely than others, for example the chromosome truncation was only observed when there was an internal TG that could seed a new telomere. We don’t think that viability is a large factor though, since all these rearrangements are tracked in an extra, non-essential chromosome VII. Either way the likelihood of each event isn’t a part of the conclusion we are trying to make, only that a variety of events are possible.

22. Line 504: Please state that no essential sequences are present telomere proximal to the break. This is obvious but would be helpful for the general reader.

There are essential sequences present telomere proximal to the break. The major advantage of our model is that it is a disome with two copies of chromosome VII, therefore the entire chromosome can be lost without losing viability. We add a note in the text to remind the readers of this feature (line 559).

23. Line 505: Maintain tense. Rewrite as” ….in fact be a feature of unstable chromosomes.”

We removed the our speculation about the potential connection between our results as the *taz1* data, and therefore deleted this sentence entirely (line 563).

24. Line511: Omit last sentence of the Discussion. The sentence ends the Discussion with an uncertain and not fully relevant issue.

We agree and deleted the last sentence.

25. Line 512: Paschini contribution can be thanked at this point or in acknowledgements.

Paschini’s contribution is thanked in the acknowledgements (line 714).

26. Lines 545-547: Please rewrite. The description is difficult to read.

We rewrote the description (lines 595-596).

27. Line 556: Replace with: “…integration of the construct….”

We corrected this typo.

28. Please do not use noc in the text. Write out word or define abbreviation.

We agreed and removed “noc” from the text, we define the abbreviation when it is used in figure legends.

29. Line 653: Replace “minimum” with “at least”

We agree and made the change (line 571-572).

30. Line 657. The In-gel hybridization is mixed with the concurrent Southern blotting please. Please rewrite to clarify.

We reorganized the in-gel hybridization and pulse-field gel protocols to not repeat the same method twice (the Southern protocol; lines 709-712).

31. Were all plasmids obtained from the outside verified?

Yes, all outside plasmids were verified via restriction digests and PCR.

Reviewer #3: In this MS, Langston et al use a strain carrying an extra Chr12 VII with a CAN1 gene located at about 25 kb from the left telomere to study by genetic means the rearrangements of the extra chromosome. This system allows distinguishing several types of rearrangements caused by chromosomal replication errors in a given genetic context. They analysed these rearrangements at permissive or semi-permissive temperature in a mutant strain carrying the cdc13F648S ts allele. Importantly, recombinant ChrVII (Class 1) can be distiguished from unstable ChrVII or an outcome of the unstable ChrVII called class 2 recombinant.
The authors found that the cdc13F684S mutant exhibit an increased of the frequency of the three types of rearrangements in contrast of the stn1T223A,S250A mutant that behaves as the WT. Their genetic analyses based on the presence of genetic markers demonstrate that the Class 1 recombinant is probably linked to replication errors occurring in or near the telomere. In contrast, insertion of internal TG1-3 tracks at distant sites from the telomere (at which the G-rich strand is replicated by the lagging polymerase) does not generate instability. They further show that inactivation of Cdc13F684S in S phase (but not in G2/M) is at the origin of the instability and is associated with extensive ssDNA in telomeres. Interestingly, cells subjected to HU and then inactivated for Cdc13 exhibit a very high frequency of unstable chromosomes indicating a synergistic effect between Cdc13 inactivation and HU-induced depletion of dNTP. They further discovered that inactivation of Exo1, Sae2, and Pif1 suppress the formation of unstable chromosomes in favour of Class 1 recombinant. While the Class 1 recombinant generated in the cdc13F684S mutant is dependent on rad52Δ, the unstable chromosome does not depend on either Lig4 or Rad52 raising the question of the mechanism generating Class2 rearrangements.

This is a solid, smart, and interesting paper. There is not much to say on the data that are mainly rigorous genetic analyses. The overall conclusion that defective Cdc13 leads to a defect associated to the passage of the replication fork at or near telomeres further processed by Exo1 is important and of general interest. The fact that this defect is at the root of the simple or complex chromosomal rearrangements is also of general interest.

I propose some suggestions or slightly different interpretations that may improve the manuscript which is by essence not very easy to follow.


1) Could the authors make a scheme at the beginning of the MS for the broad readership of PloS Genetics explaining how the CAN marker could be lost.

We reworked Fig 1B so that it’s less information dense and therefore more clear on how the CAN marker is lost.

2) The fact that the stn1T223A,S250A which has lost the ability to associate with Cdc13 does generate chromosomal rearrangements rather indicates that Stn1 is dispensable for the replication role of Cdc13. The phenotype of the double mutant may be explained by the fact that the Cdc13F684 could be less stable in the context of the stn1T223A,S250A

We reason that the stn1 mutation alone does not fully impair Cdc13 and Stn1 function, we reworded the conclusion in the text to make this more clear (as suggested by Reviewer 1 question 1, lines 177-180).

3) Explain what do you mean by replication-dependent chromosome capping

By “replication-dependent chromosome capping” we mean “whatever Cdc13 is doing at the telomere during S phase”. We remove “replication-dependent capping” in lines 272-273 and instead say “…lagging strand synthesis in S phase” because the distinction between replication-dependent and replication-independent capping was not the point of this conclusion.

4) The fact that defective Cdc13 generates chromosome rearrangements at telomere and not at TG repeats inserted internally could be due to the rescue by another converging replication fork. This rescue would be only possible at internal TG repeats and not at telomeres.

This is an excellent point and we incorporated it into our conclusions (lines 218-220).

5) Based on the absence of instability originating from the interstitial TG repeat, the authors propose that Cdc13 acts from the end of the chromosome rather than at the replisome. This argument is not maybe so strong (see point 4). In addition, Faure et al (Mol Cell 2010) showed that Cdc13 binds to the lagging telomere in a replication dependent way giving strength to the other hypothesis. The RPA like role of Cdc13 could explain the replication errors at or near the telomeres. This possibility should be at least discussed and the Faure Mol Cell paper cited.

The Faure paper was cited in the introduction on line 74. See question 4 for how we talked about Cdc13 and replication errors at or near the telomere.

6) Could the synergistic effect between Cdc13 inactivation and HU could be linked to the inhibition of late origin firing mediated by Mec1-Rad53? The synergistic effect somehow argues for a role of Cdc13 at the forks.

We agree that there’s probably a role for Cdc13 at the replication fork (see Reviewer 1 question 4). We also briefly discuss the role of defective late-origin firing on lines 496-498.

7) Hardy et al. Nature Comm (2014) showed that Sae2 and Sgs1 prevents accumulation of ssDNA at telomeres. This could be the simplest explanation to explain the role of Sae2. This paper should be cited.

We find that the *sae2Δ* rescues chromosome instability in *cdc13-F684S*, so we find it unlikely that removing Sae2’s role in preventing accumulation of ssDNA in the telomeres is responsible for the rescue. If Sae2 preventing ssDNA accumulation were involved, we would expect an increase in unstable chromosomes, not a decrease. We still cited the Hardy et al. Nature Comm (2014) paper on line 365 to account for the different roles Sae2 has at the telomere.

8) I guess Class 1 rearrangements arise by Break Induced Replication, explaining the role of Pif1 in the generation of these rearrangements

Yes, based on our genetics we think that BIR, in which Pif1 has a role, is a major player in the formation of both Class I and Class II recombinants. We think that Class I and II recombinants are likely formed by similar mechanisms, the difference being when they form; Class I early in the lineage of unstable chromosomes, and Class II later in the lineage of unstable chromosomes.

9) Are Class 2 rearrangements depend on alternative-NHEJ?

We cannot fully rule out alt-NHEJ, though we find it unlikely that it’s responsible for Class II rearrangements. While mechanisms of alt-NHEJ probably exist in *S. cerevisiae*, it’s unclear whether alt-NHEJ has a dedicated pathway, or if it is an extension of canonical NHEJ. For example there’s conflicting evidence whether alt-NHEJ depends on Lig4, the main factor of NHEJ we test in this study. However, there is agreement that the MRX complex is required for alt-NHEJ (probably to initiate short-range resection), and our deletion of Xrs2 drastically increases instability of *cdc13-F684S*.

10) The model is too complicated and should be simplified

We simplified the model (Fig 7)
